# Review: Effect of drugs on human cough reflex sensitivity to inhaled capsaicin

**DOI:** 10.1186/1745-9974-8-10

**Published:** 2012-11-12

**Authors:** Peter V Dicpinigaitis

**Affiliations:** 1 Albert Einstein College of Medicine and Montefiore Medical Center, Bronx, NY, USA; 2 Einstein Division/Montefiore Medical Center, 1825 Eastchester Road, Bronx, NY, 10461, USA

**Keywords:** Cough, Capsaicin, Antitussive, Respiratory tract infection, Asthma

## Abstract

Capsaicin, the pungent extract of red peppers, has been used in clinical research for almost three decades. Capsaicin has gained favor as the provocative agent of choice to measure cough reflex sensitivity, as it induces cough in a safe, reproducible, and dose-dependent manner. One of the major uses of capsaicin cough challenge testing has been to evaluate the effect of a pharmacological intervention on the human cough reflex. The current review summarizes the published experience with capsaicin inhalation challenge in the evaluation of drug effects on cough reflex sensitivity. A notable contrast evident between studies demonstrating a drug effect (inhibition of cough reflex sensitivity) and those that do not, is the predominance of healthy volunteers as subjects in the latter. This observation suggests that subjects with pathological cough, rather than normal volunteers, comprise the optimal group in which to evaluate the effect of potential antitussive agents on human cough reflex sensitivity.

## Introduction

Capsaicin, the pungent extract of red pepper (capsicum), has gained widespread use as a research tool among clinical investigators, as it induces cough in humans in a safe [1], dose-dependent, and reproducible manner [2,3]. Capsaicin cough challenge in humans was first described in 1984 [4], and has since been used to evaluate the effect of numerous pharmacological agents on cough reflex sensitivity. Although many drugs have been shown to inhibit induced cough in the laboratory, others have failed to do so, including agents widely regarded as clinically effective antitussives.

## Methods

A United States National Library of Medicine (PubMed) search was performed in September, 2012 using the search terms “cough” and “capsaicin” limited to human studies published in English. The abstracts of the 328 articles meeting those search criteria were reviewed and 56 studies were identified in which capsaicin cough challenge was employed to assess the effect of a pharmacological intervention on cough reflex sensitivity. Studies in which a positive drug effect was demonstrated (n = 33) are listed in Table 1[5-37]; trials in which no effect was noted (n = 30) are summarized in Table 2[4,10,12,17-19,35,37-59]. In seven of these studies, multiple drugs and/or multiple subject groups were evaluated, resulting in both positive and negative results in terms of assessment of drug activity. As the purpose of this review was to assess drug trials in which a potential therapeutic (antitussive) effect of a drug was being evaluated, studies demonstrating enhancement of cough reflex sensitivity by angiotensin-converting enzyme (ACE) inhibitors or other agents were excluded.

**Table 1:** Drugs shown to inhibit cough reflex sensitivity to capsaicin

**1**^**st **^**author**	**Ref. #**	**Year**	**Drug**	**Subject population**
Wise P	[5]	2012	menthol	healthy volunteers
Takemura M	[6]	2012	montelukast	cough-variant asthma
Ekstrand Y	[7]	2011	inhaled steroids	asthma
Ishiura Y	[8]	2010	etodolac	sinobronchial syndrome
Ishiura Y	[9]	2009	etodolac	asthma
Dicpinigaitis P	[10]	2009	guaifenesin	viral URI
Davenport P	[11]	2009	nicotine	healthy smokers
Dicpinigaitis P	[12]	2008	tiotropium	viral URI
Ishiura Y	[13]	2008	suplatast	atopic cough
Ferrari M	[14]	2007	omeprazole	asthma + GERD
Usmani O	[15]	2005	theobromine	healthy volunteers
Shioya T	[16]	2004	epinastine	atopic cough
Dicpinigaitis P	[17]	2003	guaifenesin	viral URI
Ishiura Y	[18]	2003	carbocysteine	asthma
Ishiura Y	[19]	2003	seratrodast	chronic bronchitis
Shioya T	[20]	2002	suplatast	cough-variant asthma
Dicpinigaitis P	[21]	2002	zafirlukast	cough-variant asthma
Ceyhan B	[22]	2002	oxolamine	COPD
Dicpinigaitis P	[23]	2000	baclofen	cervical SCI
Brightling C	[24]	2000	budesonide	eosinophilic bronchitis
Dicpinigaitis P	[25]	1998	baclofen	healthy volunteers
Shioya T	[26]	1998	azelastine	asthma
Dicpinigaitis P	[27]	1997	baclofen	healthy volunteers
Shioya T	[28]	1996	azelastine	cough-variant asthma
Fujimura M	[29]	1995	indomethacin	asthma, chronic bronchitis
Hargreaves M	[30]	1995	sodium cromoglycate	ACE-inhibitor cough
Hansson L	[31]	1994	lignocaine	healthy volunteers
Van Wyck M	[32]	1994	glycopyrrolate	ACE-inhibitor cough
Cazzola M	[33]	1993	theophylline	ACE-inhibitor cough
Foster G	[34]	1991	sulindac	healthy volunteers
McEwan J	[35]	1990	sulindac	ACE-inhibitor cough
Choudry N	[36]	1990	lignocaine	healthy volunteers
Fuller R	[37]	1988	codeine,morphine	healthy volunteers

Abbreviations: *URI*-acute upper respiratory tract infection; *GERD*-gastroesophageal reflux disease; *SCI*-spinal cord injury; *ACE*-angiotensin-converting enzyme.

**Table 2:** Drugs shown not to inhibit cough reflex sensitivity to capsaicin

**1**^**st **^**author**	**Ref. #**	**Year**	**Drug**	**Subject population**
Ryan M	[38]	2012	gabapentin	chronic cough
Yousaf N	[39]	2010	erythromycin	chronic cough
Dicpinigaitis P	[10]	2009	benzonatate	viral URI
Dicpinigaitis P	[12]	2008	tiotropium	healthy volunteers
Davenport P	[40]	2007	codeine	healthy volunteers
Dicpinigaitis P	[41]	2003	fexofenadine	healthy volunteers
Dicpinigaitis P	[17]	2003	guaifenesin	healthy volunteers
Ishiura Y	[18]	2003	ambroxol	asthma
Ishiura Y	[19]	2003	pranlukast	chronic bronchitis
Dicpinigaitis P	[42]	2001	celecoxib	asthma
Fujimura M	[43]	2000	mexiletine	healthy volunteers
Dicpinigaitis P	[44]	1999	zafirlukast	asthma without cough
Capon D	[45]	1996	dextromethorphan	healthy volunteers
Hansson L	[46]	1994	nicotine	healthy nonsmokers
Hutchings H	[47]	1994	codeine	healthy volunteers
O’Connell F	[48]	1994	clonidine	healthy volunteers
Fujimura M	[49]	1993	procaterol	asthma, chronic bronchitis
Choudry N	[50]	1993	MAO inhibitors	healthy volunteers
Stone R	[51]	1993	5-HT (serotonin)	healthy volunteers
Fujimura M	[52]	1992	procaterol	healthy volunteers
Ventresca P	[53]	1992	furosemide	healthy volunteers
Karlsson J	[54]	1992	furosemide, HCTZ	healthy volunteers
Studham J	[55]	1992	terfenadine	healthy volunteers
Choudry N	[56]	1991	inhaled mu opioid agonist	healthy volunteers
Smith C	[57]	1991	salbutamol, ipratropium	healthy volunteers
Choudry N	[58]	1991	granisteron (5-HT3)	healthy volunteers
McEwan J	[35]	1990	sulindac	idiopathic cough
Hansson L	[59]	1988	nedocromil	healthy volunteers
Fuller R	[37]	1988	inhaled opiates	healthy volunteers
Collier J	[4]	1984	sodium cromoglycate	healthy volunteers

Abbreviations: *URI*-acute upper respiratory tract infection; *MAO*-monoamine oxidase; *HCTZ*-hydrochlorothiazide.

## Discussion

This review has identified 33 studies in which a pharmacological intervention was demonstrated to inhibit cough reflex sensitivity to inhaled capsaicin in a variety of subject populations, thus supporting the role of cough challenge as a useful clinical tool in the evaluation of potential antitussives [3]. A striking difference between the studies showing a positive drug effect (Table 1), and those failing to demonstrate a change in cough reflex sensitivity (Table 2) is the predominant subject populations studied. Of the negative studies, 70% involved evaluation of healthy volunteers. Among the trials displaying a positive drug effect, only 27% evaluated healthy volunteers, while the majority (73%) investigated various forms of pathological cough. Of note, multiple agents were shown to inhibit cough reflex sensitivity in pathological cough, while having no effect in healthy volunteers, including guaifenesin [10,17] and tiotropium [12] in cough due to acute viral upper respiratory tract infection (URI; common cold). The leukotriene receptor antagonist zafirlukast inhibited capsaicin-induced cough in subjects with cough-variant asthma [21], but not in stable asthmatics without cough and healthy volunteers [44]. Interestingly, gabapentin has recently been shown to improve cough-specific quality of life in patients with refractory chronic cough, without affecting cough reflex sensitivity [38]. This particular study highlights the concept that the optimal approach to the evaluation of a potential antitussive agent should be multifaceted, with cough reflex sensitivity measurement complementing other measures, such as objective cough counting and subjective symptom-based questionnaires.

Conspicuous in their absence from the list of agents having demonstrated the ability to inhibit cough reflex sensitivity to capsaicin during URI are codeine and dextromethorphan, two of the most commonly used agents worldwide for the treatment of cough due to the common cold [60]. The only agents demonstrating the ability to inhibit cough reflex sensitivity to capsaicin in healthy volunteers were theobromine [15], baclofen [25,27], inhaled lignocaine [31,36], sulindac [34], systemic opiates [37], menthol [5] and, in healthy smokers, nicotine [11]. Interestingly, this list includes drugs thought to be centrally acting antitussives, as well as agents whose cough-inhibiting properties are presumed to occur through a peripheral mechanism.

Limiting the evaluation of a potential modulator of cough reflex sensitivity to a study group of healthy volunteers, whose cough reflex is not hyperresponsive, may not allow the drug to demonstrate its inhibitory effect. Thus, subjects with pathological cough appear to comprise the optimal study population when evaluating the effects of a potential antitussive agent on cough reflex sensitivity. The particular type of pathological cough best suited for evaluation of a novel antitussive may depend on the specific pharmacological action of the drug, and currently remains a question under vigorous debate.

## Competing interests

The author declares that he has no competing interest.
